# Design of Superparamagnetic Nanoparticles for Magnetic Particle Imaging (MPI)

**DOI:** 10.3390/ijms140918682

**Published:** 2013-09-11

**Authors:** Yimeng Du, Pui To Lai, Cheung Hoi Leung, Philip W. T. Pong

**Affiliations:** Department of Electrical and Electronic Engineering, the University of Hong Kong, Hong Kong; E-Mails: ymdu@eee.hku.hk (Y.D.); laip@eee.hku.hk (P.T.L.); chleung@eee.hku.hk (C.H.L.)

**Keywords:** MPI, superparamagnetic nanoparticles, medical imaging

## Abstract

Magnetic particle imaging (MPI) is a promising medical imaging technique producing quantitative images of the distribution of tracer materials (superparamagnetic nanoparticles) without interference from the anatomical background of the imaging objects (either phantoms or lab animals). Theoretically, the MPI platform can image with relatively high temporal and spatial resolution and sensitivity. In practice, the quality of the MPI images hinges on both the applied magnetic field and the properties of the tracer nanoparticles. Langevin theory can model the performance of superparamagnetic nanoparticles and predict the crucial influence of nanoparticle core size on the MPI signal. In addition, the core size distribution, anisotropy of the magnetic core and surface modification of the superparamagnetic nanoparticles also determine the spatial resolution and sensitivity of the MPI images. As a result, through rational design of superparamagnetic nanoparticles, the performance of MPI could be effectively optimized. In this review, the performance of superparamagnetic nanoparticles in MPI is investigated. Rational synthesis and modification of superparamagnetic nanoparticles are discussed and summarized. The potential medical application areas for MPI, including cardiovascular system, oncology, stem cell tracking and immune related imaging are also analyzed and forecasted.

## 1. Introduction

Over the last decade, medical imaging has been playing an important role in routine clinical practice and become indispensable for the diagnosis of a variety of diseases. Medical imaging technologies are built on different theories, and different imaging technology is preferred for different applications. Despite the great value of present medical imaging modalities for providing important diagnostic information, they still have certain limitations hampering their clinical applications. Recently, a novel medical imaging technology, called magnetic particle imaging (MPI), was invented [1]. MPI maps the distribution of magnetic tracer materials and provides advantages over the present imaging modalities. Table 1 compares the most prominent medical imaging technologies and MPI from different aspects. As it was theoretically predicted that MPI can quantitatively image tracers with acquisition time <0.1 s and spatial resolution <1 mm, high quality real-time imaging is possible [2]. Additionally, MPI does not crucially depend on magnetic field homogeneity, as in magnetic resonance imaging (MRI), so the cost to produce and maintain the magnetic field is lower. It should be noted that, as an emerging technique, there is still plenty of room for MPI to even further optimize its imaging performance and enhance its biosafety.

Magnetic nanoparticles with dimensions smaller than a critical diameter, *D*_sp_, are superparamagnetic at room temperature [3]. The magnetic anisotropy energies of superparamagnetic nanoparticles are smaller than their thermal energies, and thus, their magnetic moments are able to freely flip in any direction. On the other hand, superparamagnetic nanoparticles exhibit a nonlinear magnetization curve with no hysteresis or coercive field [3–5]. In order to prevent aggregation and chemical erosion, surface coatings are needed when synthesizing these nanoparticles [6]. The coated nanoparticles can be further functionalized with biomolecules for various biological applications [7–9]. Up until now, superparamagnetic iron oxide (SPIO) nanoparticles were most frequently investigated for biomedical applications, because they are generally stable under air and highly biocompatible [10,11]. The coated SPIO nanoparticles have been investigated for a large range of biomedical applications, such as hyperthermia, targeted drug delivery and molecular and cell separation [12–14]. Moreover, MRI contrast agents based on SPIO nanoparticles have become commercially available and can be acquired off-the-shelf [15–17].

As a tracer-based technology, MPI researches have been focused on both scanner hardware and tracer materials. Currently, SPIO nanoparticles are widely studied as MPI tracer materials because of their compelling superparamagnetic property and safe clinical history. The diameter, size distribution and surface coating are all important factors determining the biomedical performance of SPIO nanoparticles in MPI. In a recent paper, Saritas and coworkers pointed out that the MPI spatial resolution depends on the properties of the tracer material and the external magnetic field. They also foresaw that, with improvements of hardware and tailored tracers, MPI will be able to produce images exhibiting sub-mm resolutions and micromolar-level sensitivity within the next couple of years [18]. Furthermore, the rational design of SPIO nanoparticles can offer enormous potential to MPI as a powerful medical imaging modality in biomedical areas, such as cardiovascular, oncology and cell labeling [3,18,19].

## 2. Principle of MPI

**Figure 1:**
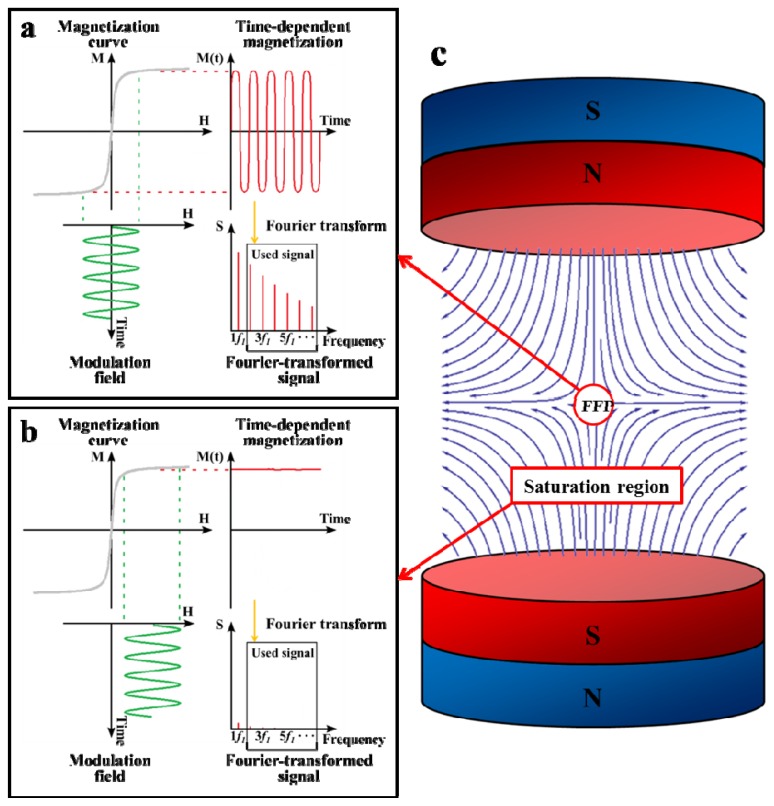
Principle of MPI configuration [19,22]. (**a**) The magnetization curve (*M*) of the superparamagnetic nanoparticles is nonlinear. When an oscillating magnetic field (modulation field) with a single frequency, *f*_1_, is applied to the superparamagnetic nanoparticles, the resulting magnetization, *M* (*t* ), is time-dependent and exhibits higher harmonics. The Fourier-transformed signals (*S*) with higher harmonics (grey box) are used for MPI imaging. The signal at *f*_1_ is excluded, because it is difficult to isolate from the superimposed modulation field signal; (**b**) When a time-independent field is superimposed upon the modulation field, the nanoparticle magnetization is always in saturation and does not significantly respond to the modulation field. The Fourier-transformed signals (*S*) are nearly non-existent; (**c**) The selection field, covering the whole region of interest (ROI), provides a field-free point (FFP). It can be produced by two magnets in Maxwell configuration.

The concept of MPI was first introduced in 2005 by Gleich and Weizenecker [1]. The tracer, which is usually SPIO nanoparticles, exhibits a nonlinear magnetization curve described by the Langevin theory of paramagnetism [18–21]. When an external magnetic field is applied, the nanoparticles align with it, and as the external magnetic field increases beyond a certain threshold, the magnetization converges to saturation. As Figure 1a shows, when the tracer is exposed to an oscillating magnetic field with frequency, *f*_1_, it exhibits a time-dependent magnetization, *M* (*t* ), which contains the drive frequency, *f*_1_, and a series of harmonic frequencies. The oscillating magnetic field is called the “modulation field”. The whole set of harmonics contained in the Fourier-transformed signal allows a quantitative measurement of the local tracer material concentration. This inverse problem can be solved by algebraic inversion of a calibration measurement.

If the tracer is exposed to a sufficiently high constant magnetic field, as shown in Figure 1b, its magnetization becomes saturated and does not respond to the modulation field. Based on this phenomenon, during MPI imaging, a static gradient magnetic field is used as the “selection field” (Figure 1c) to cover the region of interest (ROI) for spatial encoding. A field-free point (FFP) with zero magnetic field strength is provided at the center of the selection field. The magnetic field strength gradually increases from the FFP towards the edges of the selection field. The tracer is saturated by the selection field in the whole ROI, except at the FFP. As a result, only the tracer located in the vicinity of the FFP is able to respond to the modulation field and produce MPI signals. By scanning the FFP through ROI, the distribution of the tracer can be imaged without any background signal from adjacent tissues. Furthermore, since the MPI signal can penetrate the object tissues unattenuated, MPI is able to inspect the deep regions of ROI [1].

## 3. Design of Superparamagnetic Nanoparticles for Improvement of MPI Performance

As a medical imaging modality, the performance of MPI should be assessed with respect to fundamental imaging parameters, including image contrast, spatial resolution and sensitivity [23]. Since human tissue is diamagnetic, the tracer materials are the only source of the MPI signal. The MPI images exhibit a near-perfect contrast that is ideal for detecting a tracer material with minimal background [23,24]. The magnetic moment of MPI tracers is about eight orders of magnitude larger than that of protons in MRI, so MPI can theoretically achieve higher sensitivity than MRI [20]. In MPI reconstruction technology, there is trade-off between spatial resolution and signal-to-noise ratio (SNR), and consequently, spatial resolution and sensitivity should be balanced in MPI imaging technologies [20,25]. The first MPI prototype, using commercial Resovist (Schering AG Berlin, Germany) as the tracer material, provided a two-dimensional image with a very high spatial resolution of 0.3 mm × 0.5 mm by sacrificing its sensitivity [1]. The subsequent simulation studies took both spatial resolution and sensitivity into consideration and theoretically proved the possibility of sub-mm resolution and nanomolar sensitivity [20,26,27]. However, up until now, practical experiments have only demonstrated approximately 1 mm resolution and micromolar sensitivity [28,29].

The image quality of the existing medical imaging techniques, such as MRI and X-ray computed tomography (CT), mostly depends on the image reconstruction algorithm and hardware. For MPI, the properties of tracer materials also greatly govern the resulting image quality. SPIO nanoparticles are promising tracer materials as pointed out earlier. Nevertheless, for the commercially available SPIO nanoparticle agents (e.g., Resovist and Feridex (AMAG Pharmaceuticals, Lexington, MA, USA)), it was estimated that only ~3% of the ensemble exhibits the optimal nanoparticle size and can contribute to significant MPI signals [1,23,30]. This makes Resovist and Feridex far from optimal for MPI. In view of this, size optimization of SPIO nanoparticles as MPI tracers has become a burgeoning research area.

### 3.1. Magnetization Curve of MPI Tracer Materials

The theoretical magnetization curve of superparamagnetic nanoparticles is given by the Langevin theory of paramagnetism [18–20]:

(1) $$M=M_{0}L\left(\frac{H\cdot V\cdot M_{S}\cdot \mu_{0}}{k_{B}\cdot T}\right)\;\text{with }L(\alpha )=\text{coth}(\alpha )-1/\alpha$$

where *M*_0_ is the saturation magnetization of the sample, *H* is the external field, *V* is the volume of the particle, *M_S_* is the saturation magnetization of the particle material, μ_0_ is the magnetic permeability of the vacuum, *k_B_* is the Boltzmann constant and *T* is the absolute temperature. This equation reveals that the shape of the magnetization curve depends on the volume, *V*, of the magnetic core. The volume, *V*, of spherical nanoparticles is given by $V=\frac{1}{6}\pi D_{n}^{3}$ with *D_n_* being the core diameter. Larger core volume, *V* (larger core diameter, *D_n_* ), results in steeper magnetization curve.

As the superparamagnetic nanoparticles take time to respond to the modulation field, the relaxation time (τ) for the nanoparticle magnetic moment to rotate should be assessed [3]. The relaxation of superparamagnetic nanoparticles occurs in two distinct processes: Néel relaxation (τ*_N_* ) and Brownian relaxation (τ*_B_* ) [6,31]. Néel relaxation relies on the rotating magnetization vector of the nanoparticle. It depends on the volume of the magnetic core and is independent of the surrounding environment. On the other hand, Brownian relaxation occurs by physical rotation of the entire nanoparticle. It is sensitive to the hydrodynamic volume of the nanoparticle and the fluid viscosity of the surrounding solvent [32]. In practice, nanoparticles relax by the faster of the two processes, and the effective relaxation time is determined by $\tau =\frac{\tau_{N}\tau_{B}}{\tau_{N}+\tau_{B}}$ [3,24,30,32,33]. Relaxation hinders the tracer magnetization from instantaneously responding to the scanning FFP; this relaxation effect can be neglected when the modulation field frequency, $\frac{1}{\tau }>>f_{1}$. Large nanoparticles suffer from long relaxation time, τ, and, when $\frac{1}{\tau }$ is comparable with *f*_1_, the relaxation effect must be taken into consideration for MPI imaging quality.

Besides relaxation, there is a size distribution of nanoparticles in the practical situation, and this distribution is commonly approximated by a log-normal distribution function [30,34,35]. Taking both relaxation and size distribution into consideration, the equation to describe the magnetization, *M* (*t* ), of the superparamagnetic nanoparticles in the modulation field can be modified as follows [3,30]:

(2) $$M(t)=M_{S}\int_{0}^{\infty }\left(\frac{1}{1+{\left(\omega \tau \right)}^{2}}L(\alpha \text{cos}\omega \tau )+\frac{1}{1+{\left(\omega \tau \right)}^{2}}L(\alpha \text{sin}\omega \text{t})\right)g(D_{n})dD_{n}\;\text{with }\alpha =\frac{\mu_{0}H_{0}M_{S}\pi d^{3}}{6k_{B}T}$$

where ω is the angular frequency of the applied magnetic field, *D_n_* is the diameter of the nanoparticle and *g* (*D_n_*) is the log-normal distribution function of *D_n_*. This can be considered as the superposition of the magnetizations of monosized nanoparticles weighted with the probability density. The nanoparticles with a certain optimal size dominate the high-intensity MPI signal. On the other hand, the off-sized nanoparticles do not contribute a significant MPI signal; they contribute to the total amount of tracer materials, *i.e.*, the dose of tracer. Resovist is a commercially available SPIO nanoparticle material, and it is reported to have a median diameter of 5 nm and a size distribution of σ = 0.37 [24]. It was used as an MPI tracer in the first study [1] introducing the concept of MPI. However, it was revealed that only the nanoparticles of 30 nm diameter, which represent merely 3% of the iron mass in Resovist, were responsible for the MPI signal. The 97% off-sized nanoparticles in Resovist did not contribute a significant MPI signal. Therefore, the MPI performance is degraded by a size distribution that deviates from the optimal nanoparticle size.

### 3.2. MPI Imaging Quality and Nanoparticle Properties

**Figure 2:**
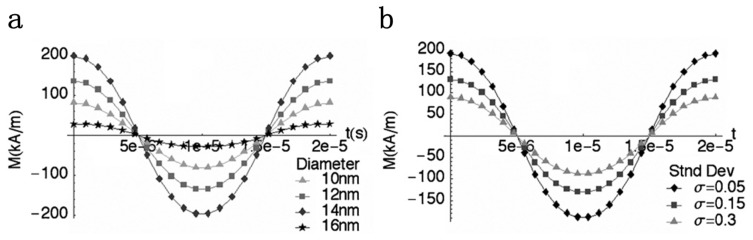
Magnetization for magnetite (Fe_3_O_4_) nanoparticles that are exposed to a 10 mT magnetic field, at 50 kHz. (**a**) Time-dependent magnetization (*M* (*t* ) ) *versus* nanoparticle diameters; and (**b**) *M* (*t* ) for increasing nanoparticle size distribution, with the median diameter being 14 nm. Reprinted with permission from [3].

The first MPI study reported that, theoretically, the MPI spatial resolution (*R* ) can be coarsely estimated by *R* = 2*H_k_/X_S_*, with *H_k_* being the alternating magnetic field (AMF) strength that makes the tracer produce substantially higher harmonics and *X_S_* being the largest spatial derivative of a selection field component [1]. Then, subsequent studies predicted the spatial resolution (*R* ) by *R* = 2*H*_1/2_/*G*, where *H*_1/2_ is the full width at half maximum (FWHM) of the derivative of the theoretical magnetization curve expressed by Equation (1) and *G* is the gradient of the selection field [19,22]. As the field strength of *H_k_* roughly corresponds to *H*_1/2_, the two estimations generally agree with each other [20]. Equation (1) explains that superparamagnetic nanoparticles with larger core diameters exhibit smaller *H*_1/2_, due to a steeper Langevin magnetization curve, resulting in better spatial resolution [19,22,36]. However, the modified Equation (2) reveals that relaxation and size distribution are also essential factors for the spatial resolution of MPI. Figure 2 shows the magnetization for magnetite (Fe_3_O_4_) nanoparticles exposed to a 10 mT magnetic field at the frequency of 50 kHz [3]. In Figure 2a, magnetization gradually increases with nanoparticle diameter before a sharp decrease, resulting from rapidly increasing relaxation time due to larger nanoparticle size. Figure 2b shows that size distribution degrades the magnetization for a given nanoparticle concentration. According to the experimental results in this study, the optimal diameter of magnetite nanoparticles is the largest diameter before the relaxation effect becomes significant, which is 14 nm in this case, while the optimal size distribution should be as narrow as possible; it is the narrowest one (σ = 0.05) in this study.

The sensitivity of an imaging modality is generally governed by SNR. SNR is related to the frequency of the modulation field, and there exists an optimum nanoparticle size at a certain modulation frequency for maximum sensitivity [3]. Generally, the optimum size is the largest nanoparticle size exhibiting a relaxation time shorter than the period of the modulation field [26]. A rigorous model study predicts that an optimum nanoparticle size for a certain modulation field with frequency *f*_0_ exists wherever the corresponding effective relaxation time, τ, approaches 1/2π*f*_0_ [30]. It is also reported that monodisperse size distribution is critical for the entire ensemble of nanoparticles to be in-phase and produce higher MPI signals, which, in turn, provides better spatial resolution and higher SNR [3,24,37]. In conclusion, both nanoparticle size (core diameter and hydrodynamic diameter), which governs relaxation time τ, and size distribution should be taken into consideration for optimal MPI spatial resolution and sensitivity. This is demonstrated by Equation (2). In practical situations, nanoparticle size is chosen by balancing spatial resolution and sensitivity or weighing one aspect for different purposes, whereas size distribution should be always as narrow as possible.

In the past several years, efforts have been devoted to designing tracer materials for optimal MPI signals in terms of image spatial resolution and imaging sensitivity [29,38]. Currently, clinical MPI scanners are still under development, and tracer design for optimal MPI performance is usually tested in MPI prototypes, which are mostly operated at ~25 kHz [24]. In a 25 kHz MPI magnetometer, magnetite (Fe_3_O_4_) nanoparticles with a 20 nm core diameter (σ = 0.26) and a 30 nm hydrodynamic diameter were adopted. They were proven to be optimal and performed with 20% better spatial resolution and four-times higher sensitivity than Resovist [29]. Recently, an iron oxide nanoparticle-micelle platform (ION-Micelle), consisting of a 25 nm (24.9 ± 1.9 nm) core encapsulated in lipidic micelles, was developed for molecular MPI purpose [38]. This material displayed a 38 nm hydrodynamic diameter and was about 200-times larger in signal than commercial SPIO agents (Endorem (Guerbet), Resovist, and Sinerem (Guerbet) in 25 kHz magnetic particle spectrometer (MPS) measurements. However, sensitivity was not determined in this study. Optimal tracer for 25 kHz MPI could be designed based on these documented results. For clinical applications, the performance of tracer materials must be able to be preserved in a biological environment and maintained consistently over a long time. During *in vivo* circulation, the superparamagnetic nanoparticles often undergo opsonization or uptake by cells. These *in vivo* reactions could slow down Brownian relaxation, which is sensitive to the hydrodynamic volume of nanoparticles. To prevent signal loss, Brownian relaxation contribution to effective relaxation time must be minimized [24]. This requires more studies on suitable surface coatings of nanoparticles.

## 4. Design of Nanoparticles for Medical Applications of MPI

MPI imaging attributes, listed in Table 1, are generally superior to those of the present medical imaging technologies. Since the MPI signal is linearly proportional to the concentration of magnetic nanoparticles, it can provide a quantitative image of nanoparticle distribution. The high temporal resolution (more than 40 volumes per second) makes MPI a promising real-time medical imaging modality [19,39]. The local interactions between modified coating nanoparticles and *in vivo* tissues suggest that MPI could be further developed for functional imaging. Generally, different medical applications require different nanoparticle properties. As previously introduced, SPIO nanoparticles are most frequently studied as MPI tracers, due to their superparamagnetic and highly biocompatible properties. Additionally, preclinical studies have been performed to investigate the design of SPIO nanoparticles for various medical areas. In this part, tracer design for medical applications of MPI is systematically reviewed and the perspective of future work is proposed.

### 4.1. Cardiovascular System

#### 4.1.1. Safe Angiography

**Figure 3:**
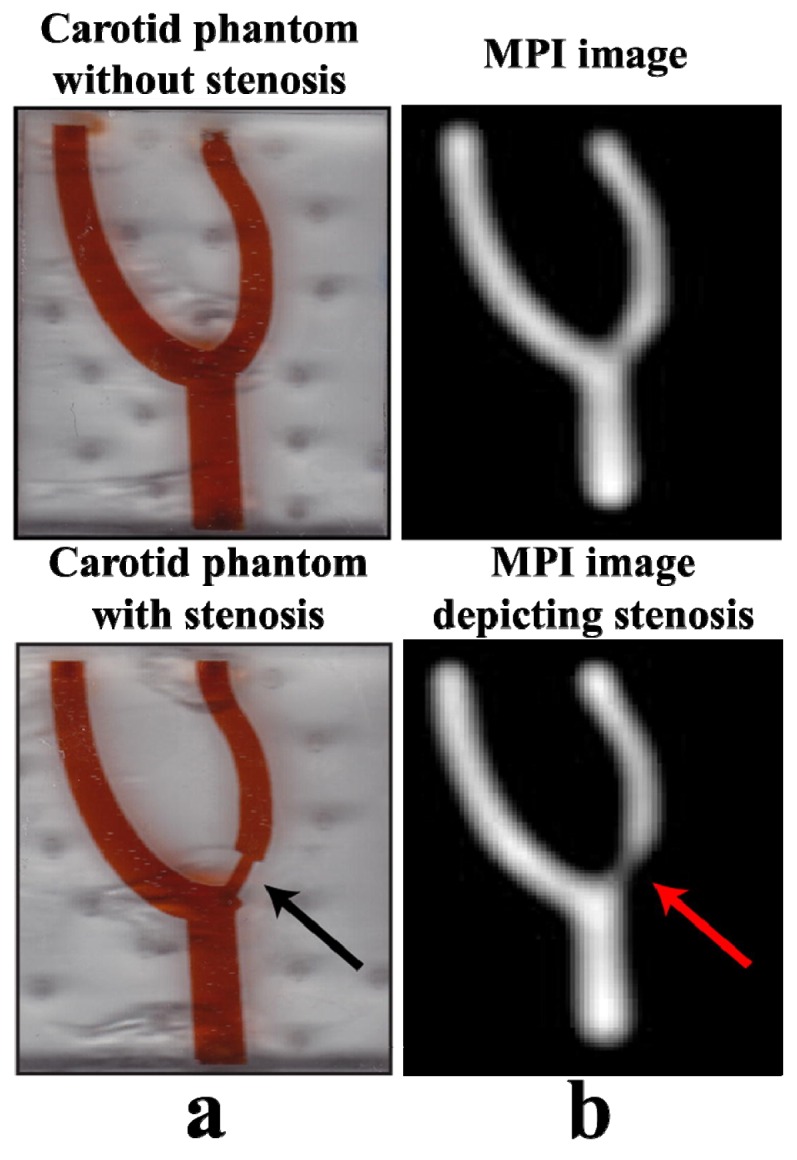
(**a**) The phantoms are used to mimic a healthy (top) and an occluded (bottom) carotid artery, and the simulating carotid arteries are filled with 20× diluted Resovist as the tracer; (**b**) Corresponding MPI images. Reprinted with permission from [18].

At present, planar X-ray angiography, CT angiography (CTA) and MR angiography (MRA) are three important angiography technologies that can provide important information for diagnosis of cardiovascular diseases. However, X-ray and CTA introduce hazardous ionizing radiation to the patients. In addition, iodine and gadolinium contrast agents used in these methods undergo renal clearance and expose patients with chronic kidney disease (CKD) to the risk of contrast-induced nephropathy (CIN) [40,41]. It is reported that about 25% of potential CTA patients have CKD, thus it is imperative to develop an alternative angiography technology for CKD patients [42–44]. The high temporal and spatial resolution and high image contrast render MPI suitable for first-pass measurements, such as dynamic angiography [19,39,45]. MPI angiography greatly increases safety and efficacy through using magnetic field and kidney-safe tracers [46,47]. Early biodistribution studies reported that iron oxide nanoparticles mostly accumulate in the reticuloendothelial system (RES) organs, and then, a commercial SPIO contrast agent, Resovist, was shown to be cleared from human body via RES instead of kidney [48,49]. As a result, SPIO nanoparticles are well tolerated in CKD patients and have been used to treat anemia in CKD patients [50]. An *in vitro* MPI study (Figure 3) used Resovist as the tracer and successfully imaged stenosis in the “carotid artery” phantoms [18]. Resovist was also applied in the first 3D *in vivo* study, and the beating heart of a mouse was detected at a temporal resolution of 46 frames per second [39]. 3D detection of the vascular system can avoid the main problems of traditional 2D cath lab, including foreshortening and overlapping [51].

For X-ray angiography, catheterized arterial injections are required to obtain a high concentration of the iodine contrast agent for the delineation of narrow blood vessels. Intravenous injection is much safer than arterial injection; however, it offers much lower contrast agent concentration. Benefiting from high contrast and high sensitivity, MPI angiography could detect low concentrations of MPI tracers, allowing the adoption of intravenous injections [23]. SPIO nanoparticles exhibiting the optimal MPI signal (reviewed in Part 3) and longer circulation time are preferred for MPI angiography. Upon intravenous injection, nanoparticles are subject to opsonization and being recognized by RES, and the circulation time is highly regulated by their hydrodynamic size, morphology, surface coating and charge [52–54]. It is generally agreed that positively charged surfaces lead to non-specific attachment to cells, while negatively charged surfaces increase liver uptake, so neural surfaces are preferred for extended circulation time [55–58]. The influences of nanoparticle surface coating, shape and flexibility are not quite clear and need more investigations [59,60]. Among all these factors, hydrodynamic size is the most critical for nanoparticle circulation and clearing. Nanoparticles smaller than kidney fenestrae (~15 nm) are swiftly cleared through kidneys, while nanoparticles larger than ~200 nm are sequestered by spleen [61–63]. It has been documented that the hydrodynamic size lying between 15 and 100 nm is optimal for longer circulation time [64–66]. In an animal (mouse) study, magnetite (Fe_3_O_4_) nanoparticles were synthesized with an oleic acid coating and subsequently transferred to the aqueous phase using a PEG-ylated amphiphilic polymer [poly(maleic anhydride-alt-1 octadecene)-poly(ethylene glycol)]. This SPIO nanoparticle material with a median diameter of 19 nm and a median hydrodynamic diameter of 86 nm in water showed a circulation time 3× longer than Resovist [24]. The biodistribution study shows that these nanoparticles accumulated in liver and spleen, thus suggesting the RES clearance routes. This tracer design is favorable to imaging quality. When detected in a 25 kHz MPS, this tracer showed a two-fold greater sensitivity and 20% better spatial resolution than Resovist.

#### 4.1.2. Red Blood Cell Labeling

Although SPIO nanoparticles exhibit longer circulation time than traditional molecular contrast agents, such as iodinated contrast media (ICM) and gadolinium-chelates, it is still not good enough for long-term monitoring. Autologous red blood cells (RBCs) have been utilized to encapsulate SPIO nanoparticles to address biocompatibility and to prolong nanoparticle circulation time [19,53,67,68]. Studies of cell loading techniques have been carried out for murine and human RBCs [68–70]. Generally, the encapsulation procedure consists of the dialysis of RBCs in nanoparticle solution and successive resealing and reannealing of dialyzed RBCs. The tracer-labeled RBCs, as a long-lasting blood pool agent, can be used to detect blood-volume-related diseases, including hemangiomas and bleedings, monitor blood supply restoration after angiography and revisit a tumor after therapy [19,71–73]. Some comparative blood volume measurements in vessel lumens can also benefit from the long-lasting homogeneous tracer concentration in blood [67].

**Figure 4:**
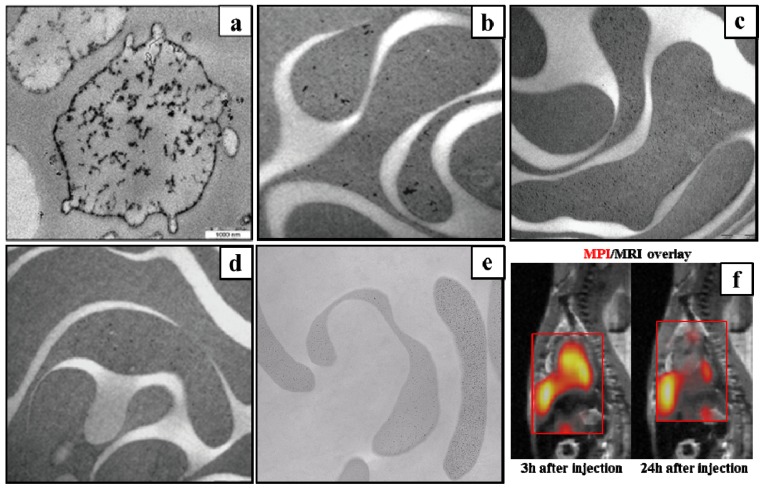
TEM image of red blood cells (RBCs) labeled by (**a**) citrate-coated superparamagnetic iron oxide (SPIO) nanoparticles; (**b**) Resovist (SHU 555A); (**c**) Sinerem (AMI 227); (**d**) PMP-50 and (**e**) P904, respectively; (**f**) MPI images (orange), displaying a sagittal slice through heart, were overlapped onto MRI reference images. MPI data was acquired 3 h and 24 h after injection of Resovist-loaded RBCs. Reprinted with permission: (**a**) from [74]; (**b**), (**c**) and (**d**) from [53]; (**e**) from [75]; and (**f**) from [67].

For efficient RBC encapsulation, both nanoparticle size and surface coating should be considered. It was reported that the hydrodynamic diameter of nanoparticles should be smaller than 60 nm for effective RBC entrapping [68]. Moreover, attachment of nanoparticles to RBC membranes should be avoided, because it would activate the elimination of RBCs by the immune system. As shown in Figure 4a, the citrate-coated SPIO nanoparticles (black dots) with a hydrodynamic diameter of 8.2 mm were effectively encapsulated into RBCs, but stuck to RBC membranes [74]. As a result, these nanoparticles are restricted for RBC labeling. On the other hand, Figure 4b–d show that three types of commercial SPIO nanoparticles, including Resovist (SHU 555A), Sinerem (AMI 227) and PMP-50, having a dextran coating and a median hydrodynamic diameter ranging 30–60 nm, were successfully loaded into human RBCs and did not bind on cell membranes [53]. A recently developed SPIO MRI contrast agent, called P904, with amino-alcohol coating and 21 nm of hydrodynamic diameter was, as well, efficiently encapsulated into human and murine RBCs and homogeneously distributed in the cell cytoplasm (Figure 4e) [75].

A recent animal experiment reported that by detecting the RBCs loaded with Resovist, MPI imaging of the blood pool of living mice was feasible several hours after injection (Figure 4f) [67]. Nevertheless, resulting from the size-selection mechanism of RBC encapsulation, the Resovist-loaded RBCs showed reduced MPS signals than the corresponding bulk tracers [67]. Novel SPIO nanoparticles, coated with dextran or amino-alcohol to prevent attachment to cell membranes, should be synthesized with a hydrodynamic diameter smaller than 60 nm and narrow size distribution. Additionally, for a specific nanoparticle size, the imaging parameters, such as the frequency of the modulation field, should be adjusted for optimal image quality.

#### 4.1.3. Atherosclerotic Plaque

Atherosclerosis is a systemic disease of the vessel wall, and vulnerable atheromatous plaque is responsible for many acute ischemic events. Atherosclerotic plaques contain various components, including connective tissue extracellular matrix, crystalline cholesterol, cholesteryl esters, phospholipids and various cells [76]. Information about the morphology and composition of plaque is essential for identifying vulnerable patients and evaluation of therapies [77]. MRI, due to its ability to perform good soft tissue contrast, has emerged as a promising technology to image atheromatous plaque and to identify plaque components [78–80]. Furthermore, molecular MRI using targeted contrast agents was developed, and it brings significant improvement to the identification of vulnerable plaques. Comparing with MRI, besides high spatial resolution, high sensitivity and high image contrast, MPI could provide valuable quantitative images to identify vulnerable plaque, given that the right amount of tracer material is accumulated.

**Figure 5:**
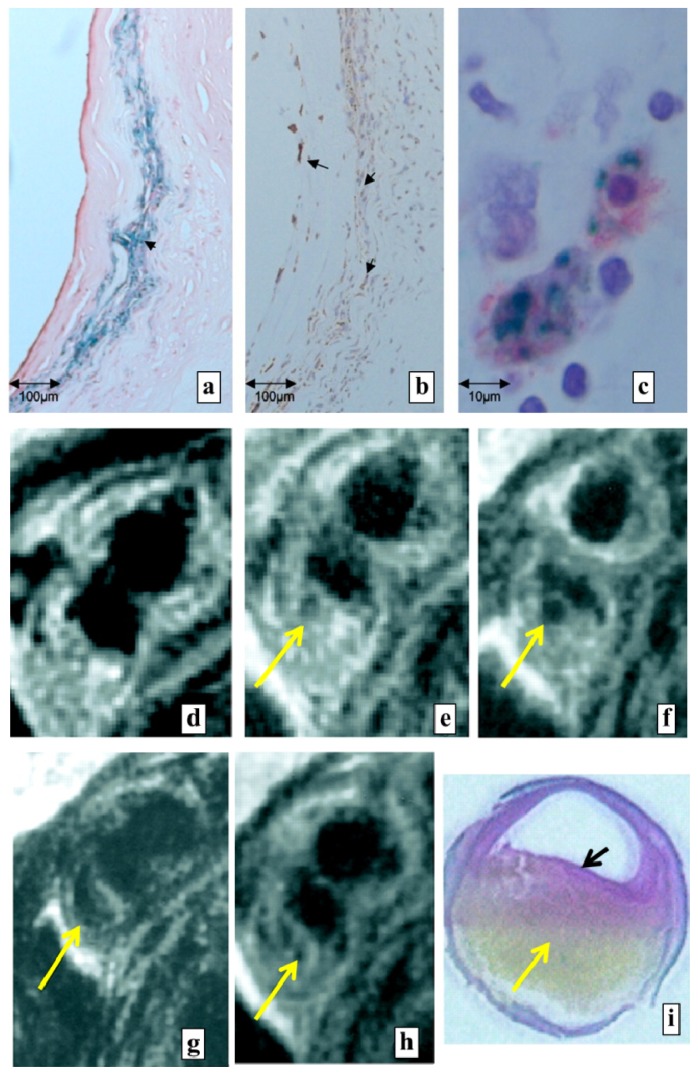
(**a**–**c**) Plaque histological sections. (**a**) Perls’ staining shows positive iron oxide accumulation (arrow; original magnification ×100) in the plaque fibrous cap; (**b**) MAC 387 stain shows the accumulation of macrophages (arrows) in the cap region of plaque (magnification ×200); (**c**) Double staining with MAC 387 antibody (red) and Perls’ reagent (blue) demonstrates colocalization of SPIO nanoparticles (Sinerem) to macrophages (magnification ×1000); (**d**–**i**) Axial MRI images show the same section of a patient’s internal carotid artery at different times. The corresponding histological section was obtained eight days after the infusion and, then, was stained with elastin van Gieson; (**d**) Before infusion of Sinerem, the fibrous cap visualized with no signal loss; (**e**) At 24 h after infusion, a signal loss area (arrow) is shown in the subendothelial region; (**f**,**g**) The size of the signal loss area has increased at (**f**) 36 h and (**g**) 48 h after infusion; (**h**) After 96 h of infusion, the signal loss area is still visible, but decreased; (**i**) The histological section of plaque shows a thin fibrous cap (black arrow) and a large lipid core (yellow arrow) that are both typical features of vulnerable plaque. Reprinted with permission from [89].

Atherosclerosis was previously considered to be a passive cholesterol accumulation in the vessel wall; however, it has now been recognized as a chronic immuno-inflammatory disease [81–83]. Several studies reported that macrophage dense inflammation was detected on or beneath the vulnerable atheromatous plaque, and this increases the risk of plaque rupture [84–86]. Some *in vivo* studies proved that the commercial MRI SPIO agents, Sinerem and Feridex, have been used successfully to label activated macrophages in vulnerable plaques, and could reveal the recruitment of macrophages into the atherosclerotic plaques to assess plaque initiation, progression and complications [87–94]. Taking Sinerem as an example, Figure 5a–c show histological sections demonstrating the colocalization of Sinerem to macrophages, and Figure 5d–h show the MRI signal changing after the administration of Sinerem. Sinerem, as well as Feridex consist of dextran coatings and undergo macrophage endocytosis, presumably through dextran receptors [95,96]. As Sinerem and Feridex both have been tested as an MPI tracer previously, it is feasible to quantitatively detect the Sinerem- and Feridex-labeled macrophages by MPI for assessment of vulnerable plaques.

Nowadays, the diagnostic procedure of cardiovascular system disease is complex. It requires different imaging technologies and may introduce adverse side effects. For example, positron emission tomography (PET) and single photon emission computed tomography (SPECT), for assessment of tissue perfusion, introduce radioactive tracers [97,98]. MPI has been studied to realize different cardiovascular applications in a safe manner, using the magnetic detection of different SPIO tracers. Additionally, simultaneous detection of multiple tracers is also undergoing progress in research, and up to three tracers have been accurately quantified [99]. Thus, a comprehensive cardiovascular MPI procedure is foreseeable. After an intravenous injection of a specific cardiovascular tracer mixture, real-time MPI angiography could be provided immediately for stenosis detection, following with the tissue perfusion image. Then, atherosclerosis tracer and tracer-labeled RBCs are imaged for atherosclerotic plaque assessment and long-term monitoring several hours or days after injection. Besides diagnostic purposes, MPI-guided intervention is feasible, as well, and instruments for visualizing SPIO-labeled intervention in MPI are under investigation [100–102]. In contrast to digital subtraction angiography (DSA), which yields 2D projections, MPI is free of ionizing radiation and could produce 3D continuous imaging of vessel trees by using long-lasting tracer labeled RBCs for more precise intervention guidance. By combining all the promising functions together with the specialized tracer, it is possible to develop a comprehensive cardiovascular MPI technology. This novel technology may offer significant reduction in complexity to the present clinical diagnostic and therapeutic procedure of cardiovascular systems.

### 4.2. Oncology

#### 4.2.1. Diagnostic Imaging

Cancer is one of the leading causes of death in the world. Enormous effort has been devoted to increase survival rates by early diagnosis and improving therapeutic methods. Among the present medical imaging technologies, MRI is most frequently used to characterize malignant tissues for its good soft tissue contrast; however, its sensitivity for distinguishing malignant tissues from normal tissues is poor. To this end, tumor staging and therapy assessment can be assisted by measuring microvascularization and monitoring blood supply [103]. These can be realized by MPI through tracer-labeled RBCs. Moreover, if targeted tracer material could specifically accumulate in tumor cells after systemic injection, MPI would realize quantitative tumor tissue measurements [104]. The development of targeted tracer materials will improve tumor staging, optimize the therapeutic plan and enable early cancer detection and assessment of therapy response.

**Figure 6:**
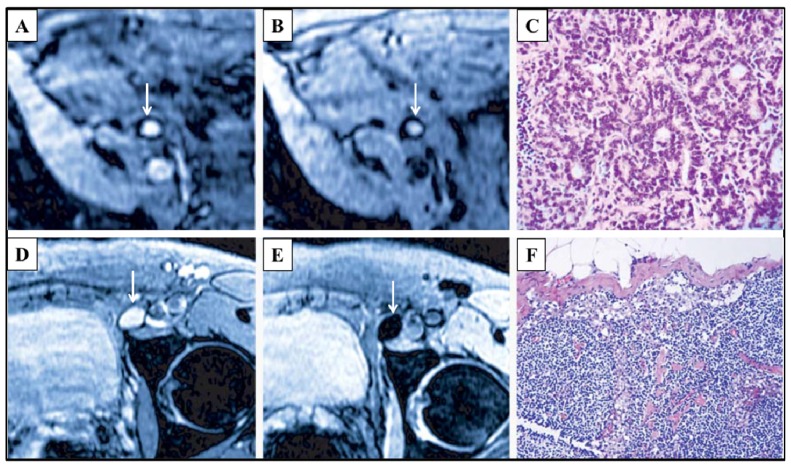
MRI images were taken from two prostate cancer patients. For one patient, an unenlarged iliac lymph node is completely replaced by tumor and presents high signal intensity in conventional MRI (arrow in Panel **A**) and Sinerem enhanced MRI (arrow in Panel **B**); The corresponding histological section (Panel **C**) (hematoxylin and eosin staining, ×200) verifies lymph node metastasis; For the other patient, a normal lymph node presents high signal intensity in conventional MRI (arrow in Panel **D**); however, it shows a homogenous signal decrease in Sinerem enhanced MRI (arrow in Panel **E**), due to the accumulation of Sinerem; Panel **F** shows the corresponding histological section (hematoxylin and eosin staining, ×200). Reprinted with permission from [114].

Tumor metastasis turns local cancer into a systematic disease, and lymphatic spread is one of the major mechanisms for tumor metastasis. In most of the malignancies, the situation of lymph node metastasis has major prognostic implications and is a major criterion for determining the necessary adjuvant chemotherapy [105–108]. Sentinel lymph nodes are the first lymphatic relays in the drainage territory of a primary tumor and are considered to be the first metastasis site in lymphatic spread [109–111]. The identification of sentinel lymph node metastasis is important for the determination of the surgery plan and estimation of prognosis. If sentinel lymph node metastasis is negative, the resection of lymph nodes could be reduced, and consequently, patients could benefit from the reduced trauma and possibly extended life expectancy. It has been reported that SPIO nanoparticles can be taken up by macrophages and then transported to lymph nodes [112,113]. As a result, the metastatic lymph nodes lacking macrophages (Figure 6a–c) can be differentiated from the normal lymph nodes that are rich in macrophages (Figure 6d–f) in SPIO-enhanced MRI images [114]. Sinerem, reported as a contrast agent specific to lymph nodes, was applied to prostate cancer and renal cell cancer patients, and lymph node metastases were successfully identified [114–117]. MPI, with the ability to directly quantify Sinerem as a tracer, is a promising alternative to identify lymph node metastasis with high spatial resolution and high sensitivity. If a tracer could be designed to specifically stick to or to be encapsulated into malignant cells, simultaneous imaging of primary tumor and tumor metastases is realizable.

#### 4.2.2. Therapeutic Imaging

Presently, surgery, radiotherapy and chemotherapy are three conventional therapy methods for cancer. However, side effects are inevitable for all these three treatments. This drives researchers to explore novel site-specific therapy methods to minimize side effects. In recent years, hyperthermia has been widely investigated based on the phenomenon that superparamagnetic nanoparticles can be heated in an AMF by the relaxation processes [26,118–120]. An animal study proved the anti-tumor effect of magnetic nanoparticles under an AMF in a mouse melanoma model [121]. The first clinical trial was carried out to treat patients with recurrence of prostate cancer [122]. In this study, hyperthermic (40–45 °C) temperature to thermoablative (>45 °C) temperature were achieved in the prostates, indicating the feasibility of magnetic-nanoparticle thermotherapy.

An important prerequisite for optimizing the treatment plan and quality control of hyperthermia is quantitative imaging of superparamagnetic nanoparticle distribution. Unfortunately, none of the present medical imaging technologies are suitable for this purpose. MRI cannot image a high concentration of superparamagnetic nanoparticles, because it would cause a signal void. CT has been investigated for visualizing deposits of nanoparticles; however, it requires multiple detections and introduces harmful ionizing radiation [122]. As previously discussed, MPI can quantitatively map superparamagnetic nanoparticle distribution. Biocompatible SPIO tracers for MPI have the potential to be adopted in hyperthermia, improving the treatment safety.

In addition to nanoparticle distribution, real-time temperature control is also important for effective hyperthermia and avoiding unwanted damage to healthy cells. Hyperthermia treatment is generally carried out between 42 °C and 46 °C [123]. Currently, real-time temperature monitoring during the hyperthermia procedure is challenging. The magnetization curve of a superparamagnetic nanoparticle is influenced by temperature. It was reported that the accurate quantification of nanoparticle temperature can be carried out in MPI by measuring the ratio of fourth/second harmonics of the magnetization [124]. Since MPI could provide comprehensive images showing the concentration and temperature of SPIO nanoparticles, it could be established as a noninvasive real-time thermometry method for thermal dosimetry of thermotherapy.

The simulation studies of magnetic fluid hyperthermia (MFH) point out that a higher heating rate requires SPIO nanoparticles with an optimized size and narrow size distribution [13,125]. The theoretical calculation indicated that, for an AMF with frequency *f*, the optimal superparamagnetic nanoparticles are those satisfying 2π*f*τ = 1, with τ being the effective relaxation time of the nanoparticles [126]. As the value of τ directly depends on the size of nanoparticle, it is crucial to optimize nanoparticle size with uniform size distribution for a certain AMF frequency. Another simulation study documented that an increase in standard deviation (σ ) of the size distribution from zero to 0.25 results in a ~85% drop in heating capacity [127]. Theoretically, spherical iron oxide nanoparticles with a diameter of ~12.5 nm offer the highest heating rate for an MFH system with the AMF frequency set at *f* = 400 kHz [125]. For an MFH system with *f* = 376 kHz, 16 nm nanoparticles were proven to be optimal, and an increase in σ from 0.175 to 0.266 results in a ~30% drop in heating capacity [126]. Similarly, 16 nm nanoparticles also optimally perform in a MFH system with *f* = 373 kHz, and a ~30% heating capacity drop resulted from an increase of σ from 0.175 to 0.284 [13]. As the requirements of SPIO nanoparticles for MFH are in agreement with the requirements of tracers for high-quality MPI, it is feasible to combine them to develop a sophisticated medical modality for real-time MPI-guided hyperthermia. SPIO nanoparticles, satisfying both effective hyperthermia and sensitive MPI temperature imaging, could be developed for such a modality.

#### 4.3. Stem Cell Tracking

**Figure 7:**
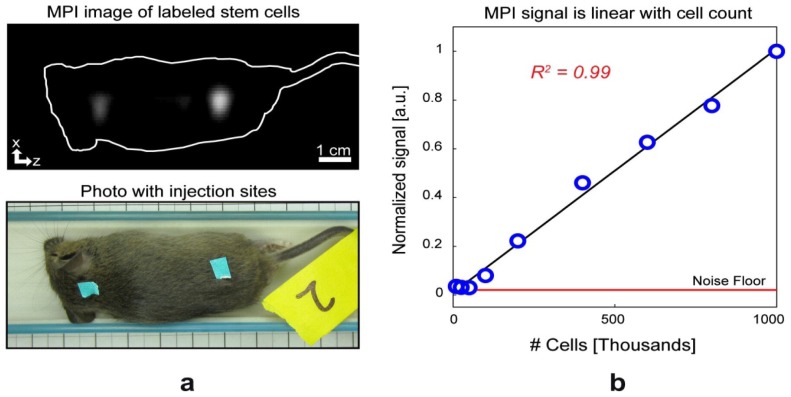
MPI imaging of tracer-labeled stem cells. (**a**) The projection MPI image shows the two subdermal injections (marked by cyan labels in the bottom photo) of hESC-derived cells into a postmortem mouse, with 1 × 10^5^ cells on the left site and 2 × 10^5^ cells on the right site. The MPI signal intensity ratio between the right and left injection regions was 2.1; (**b**) The plot of MPI signal intensity *versus* stem cell number in the scanner demonstrates a remarkable linear fit (*R*^2^ = 0.99). Reprinted with permission from [18].

Stem cells possess the capability of self-renewal and differentiation into different cell types. Stem cell implantation is a novel therapeutic strategy that is greatly promising for healing damaged organs, such as dopamine neurons in Parkinson’s disease, diabetic pancreatic cells and infarcted myocardium [128–131]. It is important to understand the *in vivo* distribution and fate of the stem cells after delivery into a patient’s body. Several current imaging methodologies have been investigated for this purpose, such as CT, MRI, PET and SPECT [132–135]. MRI, taking advantage of its good soft tissue contrast, high spatial resolution and no need for radiation exposure, gathers researchers’ attention. Detection of stem cells labeled with SPIO nanoparticles was experimented on in MRI, and the nanoparticles could be quantified in cell tracking [136]. However, this indirect quantification relies on the shift of proton magnetic resonance frequency by the magnetic fields of neighboring magnetic nanoparticles with limited sensitivity. MPI, on the contrary, directly images the distribution of tracer materials by detecting their nonlinear response of the modulation field. MPI is extremely suitable for stem cell imaging, because it images the tracer-labeled cells with high sensitivity, high contrast and nearly zero signal attenuation. Additionally, MPI is also able to accurately quantify the cell number in the imaging volume. The first *in vitro* study documents that C17.2 neural stem cells (NSCs) and rat mesenchymal stem cells (MSCs) loaded with Resovist and Feridex were detected by MPI [137]. It reveals that MPI can enable linear quantification of both iron content and cell number, and Resovist performed higher in MPI efficacy. In a recent study shown in Figure 7, two subdermal injections containing Resovist-labeled human embryonic stem cell (hESC)-derived cells were injected into a postmortem mouse [18]. The ratio of the signal intensity was roughly coincident with the ratio of the injected cells. It is feasible to track the migration of stem cells and monitor stem cell fate in the context of *in vivo* regeneration or tissue repair using MPI.

The prerequisite for effective MPI stem cell tracking is that sufficient tracer should be loaded into the stem cells and the tracer must not compromise the viability and functions of the stem cells [138]. The nanoparticles are transferred into stem cells mainly via endocytosis. The efficiency of stem cell endocytosis is strongly affected by nanoparticle properties, including size, surface charge, surface chemistry, as well as the cell line [139,140]. Generally, nanoparticles with hydrodynamic diameters smaller than ~100 nm are preferred for stem cell uptake, and positively charged surfaces are expected to facilitate endocytosis, due to the attraction with the negatively charged cell membranes [141,142]. As for surface chemistry, (carboxymethyl) chitosan-modified and citrate-modified SPIO nanoparticles were internalized into stem cells with high efficiency despite the negative surface charge [143]. Additional cell targeting functional groups can also enhance the internalization. Nevertheless, due to the complexity of the stem cell endocytosis mechanism, the parameters of a particular cell-nanoparticle system cannot be directly translated to other systems [138]. Based on the knowledge of MRI SPIO nanoparticle contrast agents for stem cell tracking, particular MPI tracers could be designed for different types of stem cells. For optimal imaging quality, the corresponding MPI parameters should be adjusted accordingly in order to facilitate monitoring and assessing of stem cell therapy.

### 4.4. Immune Related Imaging

**Figure 8:**
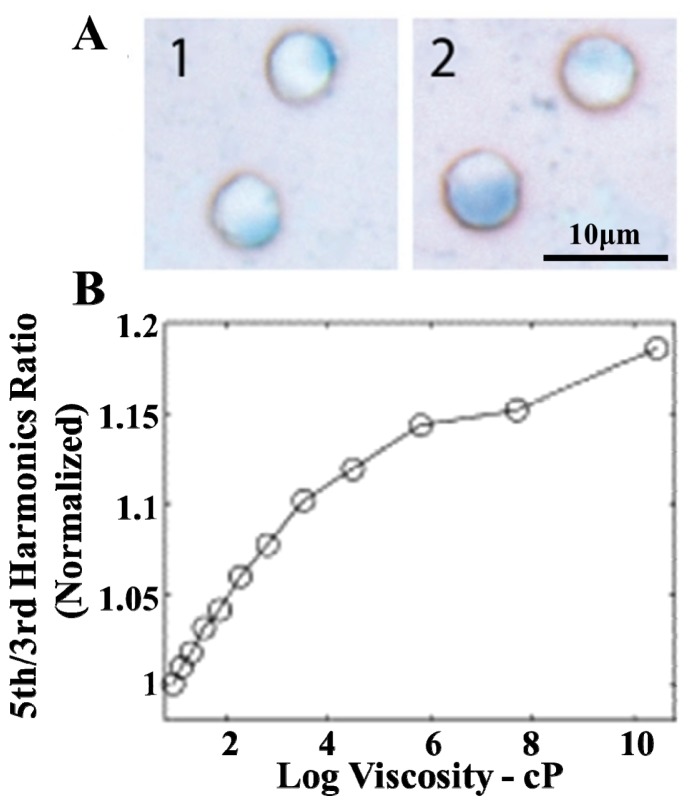
(**A**) SPIO nanoparticles diffusely distributed in T-cells after the T-cells being labeled with SPIO nanoparticles (1 mM Fe/mL) for 1 h (A1) and 4 h (A2); (**B**) Fifth/third harmonic ratio *versus* log of viscosity for Feridex dispersed in glycerol solutions with different mass concentrations. Nanoparticle excitation occurred at alternating magnetic field (AMF) (224 Hz, 10 mT). Reprinted with permission: (**A**) from [148]; and (**B**) from [149].

Besides RBCs and stem cells, immune cells can also be loaded by MPI tracers; thus, MPI may also be able to track and quantify inflammation [144,145]. As discussed in Part 4.1.3 and 4.2.1, the tracer-labeled macrophages can be applied in MPI for identifying vulnerable atheromatous plaque and metastatic lymph nodes. In general, macrophages are easy to label, because of their natural disposition to internalize particles, whereas T-cell labeling is challenging. Citrate-coated SPIO nanoparticles have been successfully loaded into T-cells [146,147]. In a recent study (Figure 8A), the citrate-coated SPIO nanoparticles were further decorated with protamine, and they achieved even better labeling efficacy in T-cells, while not affecting T-cell viability and activation [148]. T-cells play a key role in infectious and autoimmune diseases, as well as inflammatory and degenerative disorders of the central nervous system (CNS). *In vivo* T-cell tracking is of utmost interest to study the pathologies of these diseases.

The relaxation of nanoparticles is sensitive to environmental factors, such as molecule binding to the nanoparticle surface and the viscosity of the dispersion medium [18,149,150]. Binding molecules to the nanoparticle surface increases the hydrodynamic diameter of the nanoparticles, while changes of the viscosity of the dispersion fluid influence the particle rotation. Both of these effects govern the relaxation time of nanoparticles, which, in turn, can be detected by MPI. A method using the fifth/third harmonic ratio of nanoparticle magnetization is able to detect changes in viscosity (Figure 8B) [149]. It is expected to use the harmonic ratio to image reactions between functionalized nanoparticles and *in vivo* immune-related molecules or monitor local changes of viscosity due to inflammation. Magnetic-particle immune-related imaging is a huge breakthrough compared to the present *in vitro* immunoassay technologies.

## 5. Conclusions

MPI is a newly invented medical imaging technique relying on the nonlinear magnetization curve of superparamagnetic nanoparticle tracer materials. Currently, SPIO nanoparticles have been widely studied as suitable MPI tracer materials in the medical context, and their physical and biomedical properties greatly influence the performance of MPI. The provision of dedicated tracer materials is an important aspect of MPI research. It is documented that the present commercial SPIO contrast agents (e.g., Resovist) cannot provide high spatial resolution and high sensitivity when applied in MPI. SPIO tracers were designed having the optimum diameter and monodisperse size distribution. These tracers exhibited superior MPI performance in MPI prototypes. The specificity and high sensitivity of MPI make it promising for functional imaging. SPIO nanoparticles with suitable sizes and functionalized coatings are key components for MPI. A wide variety of potential clinical applications have been reviewed in this paper. In general, the tracers were inputted by intravenous administration. By advanced design of tracer materials for oral administration and inhalation, gastrointestinal and lung MPI imaging could be applied in the future. In 2011, a new concept, called acoustic MPI, was introduced aiming at expanding MPI into the detection of soft tissue properties [151]. Tracer materials again play a critical role in this new technique. Acoustic MPI carries out imaging by detecting the acoustic emissions caused by magnetization changes of MPI tracers, and its modality performance is greatly affected by the nanoparticle size. In conclusion, the proper architecture of the tracer greatly governs the functionalities, imaging qualities and clinical applications of MPI. The design of optimal MPI tracer materials is a very valuable research direction that deserves more attention.

## Figures and Tables

**Table 1:** Comparison of different medical imaging technologies.

Imaging technology	Principle	Spatial resolution	Acquisition time	Sensitivity	Quantifiability	Harmfulness
X-ray computed tomography (CT)	Scanning of radial projection of X-rays attenuated by the anatomical structures of the human body	~0.5 mm	~1 s	Millimolar level (contrast agent)	Yes	Ionizing radiation (X-ray)
Magnetic resonance imaging (MRI)	Registration of nuclear (water protons in human tissues) magnetic resonance	~1 mm	1 s–1 h	Millimolar level (contrast agent)	Yes (but require complex mathematical methodology)	Bio-effects caused by magnetic field
Positron emission tomography (PET)	Coincidence detection of two γ-quanta generated from the positron emission of radioactive tracers	~4 mm	1 min	Picomolar level (radioactive tracer)	Yes	Ionizing radiation (β/γ radiation)
Single photon emission computed tomography (SPECT)	Detection of the density of γ-quanta emitted by the radioactive tracers	~10 mm	1 min	Picomolar level (radioactive tracer)	Yes	Ionizing radiation (γ radiation)
Magnetic particle imaging (MPI)	Detection of the nonlinear response of superparamagnetic nanoparticles to the oscillating magnetic field	<1 mm	<0.1 s	Micromolar level (magnetic tracer)	Yes	Bio-effects caused by magnetic field
